# Pruning-fruit retention and plant models strategies to improve growth, quality, and yield of netted melon in Arid regions

**DOI:** 10.1371/journal.pone.0341551

**Published:** 2026-03-13

**Authors:** Wei Song, Xinchao Ma, Zhanming Tan, Xuefei Guo, Wanrong Wang, Turdi Yimingjiang, Xiaoqin Yao, Xiangbo Liu

**Affiliations:** 1 Xinjiang Production & Construction Corps Key Laboratory of Facility, Tarim University, Alar, China; 2 Agricultural Science Research Institute of the 3rd Division, Xinjiang Production & Construction Corps, Tumushuke, China; University of Education, PAKISTAN

## Abstract

Netted melon (*Cucumis melo var. reticulatus*) exhibits strong environmental adaptability and high economic value, making it a widely favored horticultural crop in local markets. In the arid regions of Northwest China, optimizing traditional monoculture planting patterns can fully exploit the production potential of netted melon. Economic benefits and environmental adaptability can be enhanced by adjusting plant spacing and pruning-fruit retention strategies, thus promoting sustainable agricultural development in arid Gobi regions. A field experiment was conducted with two factors: plant spacing and pruning-fruit retention strategies. Three plant spacing were set at 55, 65 and 75 cm, and three pruning-fruit retention methods included two vines with two fruits, three vines with two fruits and three vines with three fruits. The effects of different planting configurations on the growth yield and fruit quality of Gobi netted melon was evaluated. A Back Propagation Neural Network-Non-dominated Sorting Genetic Algorithm II (BP-NSGA-II) model was employed to simulate and optimize planting strategies. Increasing the number of retained vines and fruits moderately delayed plant development and postponed harvesting time. Enlarged plant spacing increased vine diameter and leaf number but inhibited vine elongation and leaf area expansion. Increasing the number of retained vines suppressed vine thickening but promoted leaf number expansion, and increased fruit retention number inhibited vine diameter growth. The effect of plant spacing on yield showed a decreasing trend as plant spacing increased, whereas the three vines with two fruits treatment consistently produced higher yields than the other pruning-fruit retention methods. Increasing plant spacing improved the comprehensive fruit quality, and the three-vine two-fruit method resulted in superior overall fruit quality. By employing BP-NSGA-II multi-objective optimization, the optimal planting configuration was determined to be a plant spacing of 70 cm with a pruning-fruit retention strategy of two vines with two fruits. Under these conditions, according to the model, the optimized growth duration would be 107 days, the maximum yield would reach 48.68 t hm^-2^ and the optimized fruit quality compliance index (Ci) would be 0.80. This strategy effectively achieves early maturity, high yield and superior fruit quality, contributing to the sustainable development of agriculture in arid Gobi regions.

## 1. Introduction

Netted melon (*Cucumis melo var. reticulatus*), also known as the European melon, is a high-quality subspecies of melon renowned for its exceptional flavor and texture, making it one of the world’s most important horticultural crops [1]. Xinjiang is one of the key production areas for melons in China, with the melon planting area reaching 116,500 hectares in 2020, accounting for approximately 30% of China’s total planting area [2]. In 2020, the melon planting area reached 116,500 hectares, accounting for approximately 30% of China’s total planting area. Melon cultivation plays a crucial role in adjusting the agricultural structure of Xinjiang, promoting farmers’ income and advancing sustainable agricultural development [3]. In recent years, melon has become a dominant and characteristic industry in Xinjiang, with its planting area expanding gradually. However, melon cultivation in this typical arid region faces dual challenges of water scarcity and fragile ecological conditions, which are further exacerbated by global climate change. Therefore, melon cultivation not only needs to adapt to extreme climatic conditions but also requires high yield and quality production under limited resources.

Crop growth and development are influenced by various factors, amongst which planting pattern plays a critical role. Appropriate planting density enables crops to achieve increased leaf areas, thus maximizing solar radiation interception, which, in turn, affects crop metabolism and the progression of the growing season [4,5]. Previous studies have shown that reducing planting density increases the stem diameter, leaf length, leaf width and leaf area of melons, thereby improving fruit quality, though it resulted in a decrease in yield [6,7]. Singh et al. [8,9] observed in their studies on long melons that, regardless of cultivation conditions, the highest yield was achieved at the minimum plant spacing (50 cm × 20 cm). However, when planting density was increased to boost yield, the number of fruits per plant and individual fruit weight decreased, leading to a reduced percentage of marketable melons [10]. Moreover, melon growth characteristics at different planting densities are determined by the genotype of the melon variety [11,12]. Therefore, the planting density for the melon varieties used in the present study must be optimized to promote growth and improve yield and quality.

The yield and quality of crops are largely determined by the appropriate source–sink relationship, where the developing fruits serve as the main sink in the plant [13]. Effectively maintaining a balance between vegetative and reproductive growth, limiting stem and leaf growth and increasing the transfer of photosynthetic assimilates to the fruits can be achieved through manual thinning, thereby improving yield [14]. This process also enhances fruit flavor and internal quality [15]. Salehi et al. [16] pointed out that appropriate thinning measures significantly increase the sugar content and mineral concentrations in melons. Similar results were obtained by Deka et al. [17] in their watermelon research, where retaining a single fruit per plant significantly enhanced growth, yield and quality. Additionally, proper pruning of melon vines can improve plant ventilation and light exposure, thereby increasing photosynthetic efficiency, which, in turn, boosts growth and significantly enhances yield and quality [18,19]. Uygun et al. [20] demonstrated that a double-main vine pruning method increased melon yield by 20% compared with single-main vine pruning and improving the overall fruit quality. In melon production management, thinning and pruning techniques are commonly used together to increase yield and quality [21]. Currently, the most commonly used pruning method is a single vine with a single fruit, which is easy to manage, yields more uniform fruit and reduces pest and disease incidence [22,23]. However, this method results in lower overall yield than other methods [24]. In melon production in arid regions, determining the appropriate pruning-fruit retention strategy has become an urgent issue.

However, most current research has focused on single-factor influences, such as planting density, thinning or pruning methods, on melon growth and development, with only a few studies addressing the combined effects of these factors [25,26]. Research on how to coordinate planting density and pruning-fruit retention strategies to improve melon yield and quality is still limited. The present study aimed to clarify the effects of different planting densities and pruning-fruit retention strategies on the growth, yield and quality of netted melon. A BP-NSGA-II model was used to optimize the planting pattern for netted melon cultivation in arid regions, providing a theoretical foundation for developing efficient cultivation models and offering practical guidance for local melon production.

## 2. Materials and methods

### 2.1. Overview of the study area

**Fig 1:**
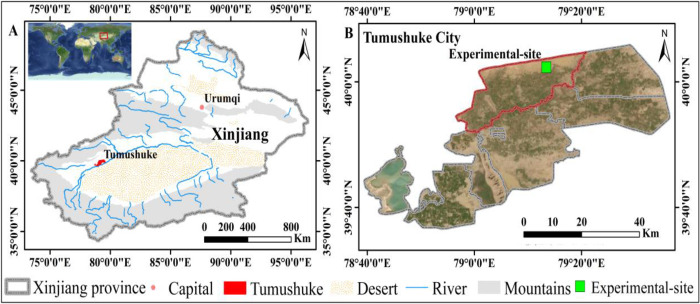
Study Area Map. Note: The standard map of China – Map Review Number GS (2020) 4619.

**Fig 2:**
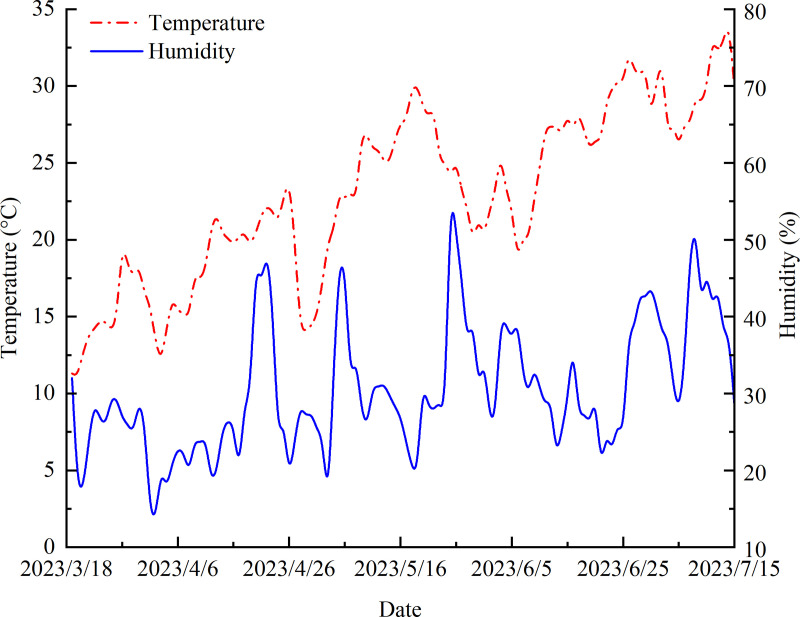
Temperature and relative humidity variations in the study area.

The experiment was conducted in 2023 at the Melon Experimental Demonstration Base in Tumushuke, Xinjiang Uygur Autonomous Region, China (79°13’44.4828′′ E, 40°00’41.5152′′ N, altitude = 1302 m; Fig 1). During the experimental period, temperature and relative humidity variations were recorded using the RR-9100 (Beijing Yugen Technology Co. Ltd., Beijing China; Fig 2). The tested melon (*Cucumis melo* L.) variety was ‘Tiandu Yinmi’, which is widely cultivated in the region due to its excellent fruit quality and adaptability to local climatic conditions.

**Fig 3:**
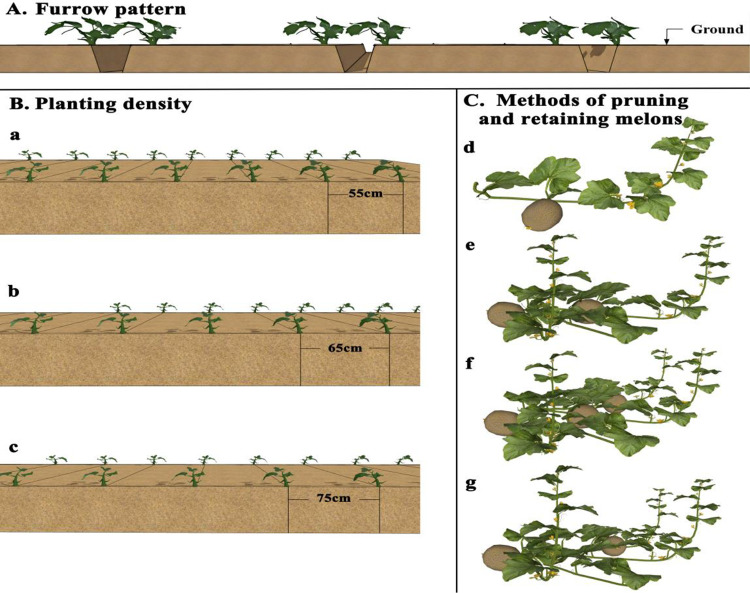
Planting pattern. **A.** Groove pattern. **B.** Plant spacing (a: 55 cm; b: 65 cm; c: 75 cm). **C.** Pruning and retention method (d. single vine and single melon. e. double vine and two melons. f. three vines and three melons. g. three vines and two melons).

The experiment was conducted using a furrow planting system, with each plot measuring 10 m × 3.2 m = 32 m². The plots were arranged in a randomized complete block design (RCBD) with protective rows surrounding each plot. Furrows were mechanically prepared and covered with plastic mulch. The furrow top width and depth were 0.5 m, with a furrow centre-to-centre distance of 3.2 m. Each furrow accommodated two rows of melon plants, with a large row spacing of 2.6 m and a small row spacing of 0.6 m. Sowing was carried out manually on March 18, 2023, as illustrated in Fig 3A. The soil in the study area is classified as sandy loam, and its physicochemical properties are presented in Table 1. The experimental field was irrigated using a sub-mulch drip irrigation system, with drippers delivering water at a flow rate of 2 L h^-1^ and spaced 0.55 m apart. Throughout the entire growth period, irrigation and fertigation were applied in accordance with the growth requirements of the melon crop. Standardized cultivation management practices were implemented across all treatments to ensure consistency.

**Table 1:** Soil physicochemical properties.

Year	pH	SOM	TN	TP	TK	AN	AP	AK
		(g kg ^− 1^)	(g kg ^− 1^)	(g kg ^− 1^)	(g kg ^− 1^)	(mg kg ^− 1^)	(mg kg ^− 1^)	(mg kg ^− 1^)
2023	8.35	11.69	0.64	0.56	4.18	83.09	14.61	363.59

### 2.2. Experimental design

The experiment was designed as a two-factor RCBD, incorporating plant spacing and pruning-fruit retention method as the two experimental factors. The plant spacing was set at 55, 65 and 75 cm, a total of three levels. The pruning and melon retention method was set up with a total of four levels: single vine and single melon, double vine and two melons, three vines and two melons and three vines and three melons. A total of 10 treatments, including a conventional cultivation mode as a control (CK), were established. The detailed experimental design is shown in Table 2.

**Table 2:** Experimental design for different plant spacing and pruning-fruit retention methods.

Treatments	CK	T1	T2	T3	T4	T5	T6	T7	T8	T9
Pruning and fruit retention method	1V1F	2V2F	3V3F	3V2F	2V2F	3V3F	3V2F	2V2F	3V3F	3V2F
Plant spacing (cm)	55	55	55	55	65	65	65	75	75	75

single vine one fruit: 1V1F; double vine two fruits: 2V2F; triple vines three fruits: 3V3F.

The single-vine single-fruit treatment did not require topping. For the double vines, two fruits, triple vines, two fruits, and triple vines, three fruits treatments, timely topping was performed when the fourth to fifth leaf of the main vine was fully expanded. Afterwards, on the basis of the number of retained vines in each treatment, 2–4 nodes of the side vines were kept, and the remaining axillary buds were periodically removed. The fruit-setting position was at the fifth to the seventh nodes of the main vine or side vines. Each main vine or side vine retained three fruits, and when the young melons reached the size of a walnut, one fruit with the most regular shape was selected for retention, whereas the other two fruits were removed. Later, timely harvesting was carried out on the basis of the maturity of the melons. The illustrations of plant spacing and pruning-fruit retention methods are shown in Figs 3B and 3C.

### 2.3. Measurement items and methods

#### 2.3.1. *Survey of the growth period.*

The growth period of the 10 treatments was recorded and investigated from the sowing of the tested melon variety to fruit maturity. The stages tracked included emergence period, vine elongation period, female flower blooming period, fruit setting period, fruit expansion period and maturity period. The number of days from sowing to fruit maturity (maturation time) and the total growth period (full growing period) were calculated.

#### 2.3.2. *Growth index determination.*

Once all treatments entered the vine elongation stage, six healthy melon plants were randomly selected and marked in each treatment. Growth parameters were measured at 7-day intervals, for a total of 10 measurements. Vine length was measured using a tape measure, from the node of the cotyledon to the furthest growing point of the plant. Vine diameter was measured at the main vine’s diameter between the first and second true leaves by using a Vernier calliper. Leaf count was determined by visual inspection, with leaves longer than 2 cm counted as mature leaves. Leaf area was measured using an LI-3000 Leaf Area Scanner (LI-COR, Nebraska Lincoln, USA), and the largest leaf from the middle-upper section of the plant was used for the measurement.

#### 2.3.3. *Measurement of yield and fruit quality.*

After the melon fruits in each treatment reached maturity, they were manually harvested. The fruits were weighed directly using a balance and the weight of a single melon was recorded to calculate the yield per hectare.

For the fruit moisture content, the peel, seeds and placenta were removed from the harvested melons. Then, five random samples of melon were weighed. The moisture content was determined using the drying method. Specifically, the fresh weight of the sample was recorded, and the sample was dried in a DHG-9625A oven (Shanghai Yiheng Scientific Instruments Co., Ltd., Shanghai, China) at 105 °C for 30 min, followed by drying at 75 °C until a constant weight was achieved. The dried weight was then recorded, and the moisture content was calculated on the basis of the difference between the fresh and dry weights [27].

For the determination of fruit shape index, five melons from each treatment were randomly selected. The longitudinal and transverse diameters of each fruit were measured using a ruler, and the fruit shape index was calculated by dividing the longitudinal diameter by the transverse diameter [28]. The peel, seeds and placenta were removed, and the thickness was measured using a Vernier calliper. The fruit hardness at the equatorial region was determined using a handheld fruit hardness tester (GY-3, Zhejiang Top Cloud Agriculture Technology Co., Ltd., Hangzhou, China) [29].

For the determination of internal fruit quality, three random samples were selected from each treatment. The soluble solid content was measured using an ATAGO-P32 handheld refractometer (ATAGO Co., Ltd., Tokyo, Japan). The soluble protein content was determined using the Coomassie Brilliant Blue G-250 method [30], the soluble sugar content was measured using the anthrone colorimetric method [31] and the vitamin C content was determined using the 2,6-dichlorophenol indophenol method [32].

#### 2.3.4. *Entropy weight TOPSIS model.*

The entropy-weight-based TOPSIS model overcomes the influence of subjective factors on the final evaluation results compared with the traditional TOPSIS model [33]. This model has significant potential for crop cultivation in arid regions [34]. An entropy-weight TOPSIS simulation was conducted for the 10 different treatments to evaluate the internal and external quality parameters of muskmelon fruits, which involve nine parameters, yielding a comprehensive quality fitting degree Ci for the fruits. The calculation steps are as follows [35,36]:

The original evaluation parameter matrix was constructed, where a refers to evaluation objects, and b refers to evaluation indicators. The original data can be written as a matrix X = (Xij)ₐ × b, where Xij represents the j-th indicator of the i-th treatment. In this experiment, a = i = 10 (number of treatments) and b = j = 9 (number of indicators). The sum of squares of the indicator was normalized to obtain the normalization matrix Y = (Yij)a × b, namely:


$${\text{Z}}_{\text{ij}}=\frac{{\text{X}}_{\text{ij}}}{\sqrt{\sum_{\text{i}=\text{1}}^{\text{a}}{\text{X}}_{\text{ij}}^{\text{2}}}}$$ (1)

The following formula was used to calculate the proportion of the i-th treatment under the j-th indicator, which was then used for computing the relative entropy probability:


$${\text{P}}_{\text{ij}}=\frac{{\text{Y}}_{\text{ij}}}{\sum_{\text{i}=\text{1}}^{\text{a}}{\text{Y}}_{\text{ij}}}$$ (2)

The entropy value Hj for the j-th indicator was calculated using the following formula:


$${\text{H}}_{\text{j}}=-\frac{\text{1}}{\text{ln}\;\text{a}}\sum_{\text{i}=\text{1}}^{\text{a}}{\text{P}}_{\text{ij}}\;\text{ln}\;{\text{P}}_{\text{ij}}$$ (3)

The entropy weight ωj for the j-th indicator was calculated using the following formula:


$$\omega_{\text{j}}=\frac{\text{1}-{\text{H}}_{\text{j}}}{\text{b}-\sum_{\text{j}=\text{1}}^{\text{b}}{\text{H}}_{\text{j}}}$$ (4)

The normalised matrix Y = (Yij)a × b was multiplied by the entropy weight ωj to obtain the weighted decision matrix Z = (Zij)a × b.


$${\text{Z}}_{\text{ij}}=\omega_{\text{j}}\times {\text{Y}}_{\text{ij}}$$ (5)

In the entropy-weighted TOPSIS model, the positive ideal solution (best vector) and the negative ideal solution (worst vector) are determined based on the maximum and minimum values of each column in the weighted decision matrix Z=(Zij)a × b.


$${\text{Z}}^{+}={(\text{Z}}_{\text{max1}},{\text{Z}}_{\text{max1}},\cdots ,{\text{Z}}_{\text{maxb}})$$ (6)

$${\text{Z}}^{-}={(\text{Z}}_{\text{min1}},{\text{Z}}_{\text{min1}},\cdots ,{\text{Z}}_{\text{minb}})$$ (7)

In the entropy-weighted TOPSIS model, the positive ideal solution (best vector) and the negative ideal solution (worst vector) were determined on the basis of the maximum and minimum values of each column in the weighted decision matrix Z = (Zij)a × b. Moreover, the distance between the i-th evaluation object and the positive ideal solution (Z+) and negative ideal solution (Z-) were calculated using the Euclidean distance formula as follows:


$${\text{D}}_{\text{i}}^{+}=\sqrt{\sum_{\text{j}=\text{1}}^{\text{b}}{\left({\text{Z}}_{\text{maxj}}-{\text{Z}}_{\text{ij}}\right)}^{\text{2}}}$$ (8)

$${\text{D}}_{\text{i}}^{-}=\sqrt{\sum_{\text{j}=\text{1}}^{\text{b}}{\left({\text{Z}}_{\text{minj}}-{\text{Z}}_{\text{ij}}\right)}^{\text{2}}}$$ (9)

The closeness coefficient (Ci) of the i-th evaluation object to the optimal solution in the entropy-weighted TOPSIS model was calculated as follows:


$${\text{C}}_{\text{i}}=\frac{{\text{D}}_{\text{i}}^{-}}{\left({\text{D}}_{\text{i}}^{+}+{\text{D}}_{\text{i}}^{-}\right)}$$ (10)

R is the goodness of fit, and the closer it is to 1 indicates the better the fit.


$$𝐑=\frac{\sum \left(x-\bar{x})(y-\bar{y}\right)}{\sqrt{\sum {\left(x-\bar{x}\right)}^{\text{2}}}{\Sigma (y-\bar{y})}^{\text{2}}}$$ (11)

x represents the independent variable, and y represents the dependent variable. References: Frost, (2019). Regression Analysis: An Intuitive Guide for Using and Interpreting Linear Models. Statistics by Jim Publishing.

#### 2.3.5. *BP-NSGA-II multi-objective optimization model.*

Firstly, a multi-objective optimization model was established using the Back Propagation (BP) neural network, leveraging its strong nonlinear interpolation capability and self-learning ability to capture the fuzzy correlation between decision variables and target variables [37]. A multi-objective optimization model was established using plant spacing and pruning-fruit retention method in netted melon cultivation as the network inputs, whereas the total growth period (days) yield and comprehensive quality fitting degree (Ci) were set as the network outputs. The BP neural network was conFigd with two hidden layers: the first hidden layer had 12 neurons, and the second hidden layer had eight neurons. The network was trained using the following parameters: number of training iterations: 2000, training function: trainlm (Levenberg–Marquardt algorithm), learning rate: 0.05, performance function: mean squared error (MSE) and training target: 1 × 10^−8^. An optimized multi-objective decision-making model was developed by conducting multiple training sessions on the neural network, effectively capturing the complex relationships amongst cultivation strategies, growth duration, yield, and fruit quality.

**Fig 4:**
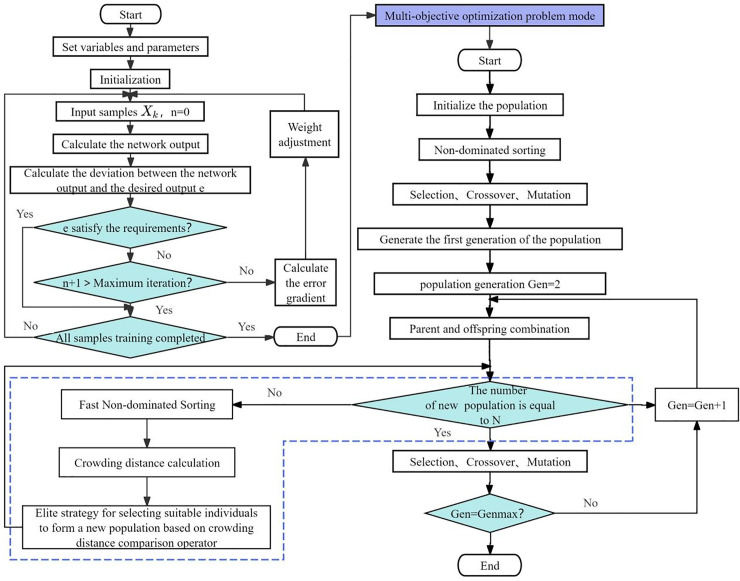
BP-NSGA-II multi-objective optimization model principle flowchart.

After the multi-objective optimization model trained by the BP neural network was obtained, the Non-dominated Sorting Genetic Algorithm II (NSGA-II) with an elite strategy was used to solve for the Pareto optimal solutions. Compared with the traditional multi-objective genetic algorithms, NSGA-II ensures a uniform distribution of solutions in the solution space maintains population diversity and offers high computational efficiency and strong robustness [38]. Owing to these advantages, NSGA-II has been widely applied in various research fields [39–41]. However, studies applying BP-NSGA-II to optimize netted melon cultivation strategies remain limited. In the present study, BP-NSGA-II was integrated with the BP neural network for optimisation and decision-making. The algorithm parameters were set as follows: total population size: 100, crossover probability: 0.8, mutation probability: 0.1 and number of iterations: 100. The working principle of the BP-NSGA-II multi-objective optimization model is illustrated in Fig 4.

### 2.4. *Data statistical analysis*

Data calculations and table creation were performed using Microsoft Excel 2010. Origin 2021 was used for plotting the graphs, and MATLAB R2023b was employed for multi-objective optimization. SPSS (version 13.0) statistical software was utilized for variance analysis, with significant differences between different treatments tested using Duncan’s multiple range test (*p* < 0.05).

## 3. Results

### 3.1. Effect of different plant spacing and pruning methods on the growth period of netted melon

As shown in Table 3, the growth periods of netted melon under different treatments varied after sowing. Before fruit set, the time differences of each growth stage amongst all treatments were within 4 days. However, after fruit set, when melons started to enlarge, the developmental differences amongst the treatments became more pronounced. CK treatment entered the swelling period first and matured the earliest, whereas T2 treatment entered the swelling period and matured the latest. The pruning method of multiple vines and fruits resulted in later entry into the swelling and maturation periods than in CK, regardless of plant spacing. Under the same plant spacing, the order of entering the swelling period was as follows: T1 > T3 > T2, T4 > T6 > T5 and T7 > T9 > T8, indicating that as the number of vines and fruits increased, the entry into the swelling period was delayed. Under the same pruning method, increasing the plant spacing caused no significant difference in the time of entry into the swelling period, with a variation of no more than 2 days. The effect of different plant spacing and pruning methods on the maturation time was similar to that on the swelling period, except that when three vines were left, the number of fruits did not affect the timing of maturity.

**Table 3:** Netted melon growth period progress.

Treatments	Seedling emergence period（date）	Vine elongation （date）	Female flower blooming period（date）	Fruit set period（date）	Fruit enlargement period（date）	Maturity stage（date）	Fruit development days（day）	Total growth period（day）
CK	2023/4/2	2023/5/1	2023/5/19	2023/5/25	2023/6/10	2023/6/26	33	101
T1	2023/4/1	2023/5/1	2023/5/22	2023/5/27	2023/6/17	2023/7/5	40	110
T2	2023/4/2	2023/5/1	2023/5/22	2023/5/28	2023/6/25	2023/7/14	48	119
T3	2023/4/1	2023/4/29	2023/5/23	2023/5/29	2023/6/23	2023/7/14	47	119
T4	2023/3/30	2023/4/30	2023/5/21	2023/5/27	2023/6/16	2023/7/4	39	109
T5	2023/3/30	2023/4/26	2023/5/21	2023/5/28	2023/6/23	2023/7/13	47	118
T6	2023/3/31	2023/4/28	2023/5/19	2023/5/27	2023/6/21	2023/7/12	47	117
T7	2023/3/31	2023/4/30	2023/5/21	2023/5/27	2023/6/16	2023/7/2	37	107
T8	2023/3/30	2023/4/30	2023/5/21	2023/5/29	2023/6/23	2023/7/13	46	118
T9	2023/3/31	2023/4/26	2023/5/20	2023/5/28	2023/6/22	2023/7/13	47	118

CK treatment had the shortest time to fruit maturity, 16 days earlier than the longest time in T2. The total growth period was the shortest in CK also, 18 days less than the longest periods in T2 and T3. For the double-vine pruning treatments, T1, T4 and T7 had fruit maturity times of 37–40 days, with a total growth period of 106–109 days. By contrast, the triple-vine pruning treatments had fruit maturity times of 46–48 days, with a total growth period of 117–119 days. Plant spacing evidently had no significant effect on fruit maturity time nor total growth period, whereas the number of vines was positively correlated with fruit maturity time and total growth period. Additionally, the number of fruits left did not have a significant impact on fruit maturity time nor total growth period.

### 3.2. Effect of different plant spacing and pruning methods on the growth of netted melon

#### 3.2.1. *Growth changes in vine length and vine thickness.*

**Fig 5:**
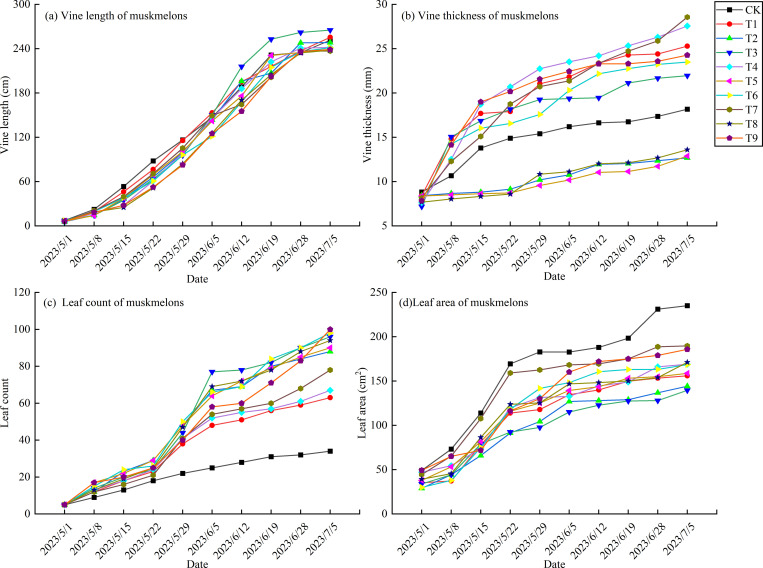
The effect of different plant spacing and pruning methods on the growth of netted melon.

As shown in Fig 5a, the vine length of each treatment followed an ‘S’-shaped growth curve, with a pattern of ‘slow-fast-slow’ changes as the growth period of the netted melon progressed [42]. Before May 15, the vine length increased slowly. After May 15, the vine length grew rapidly, and after June 19, the growth rate of the vine length in all treatments began to slow down. Throughout the entire growth period, the vine length of T3 became significantly greater than that of the other treatments starting from June 12, whereas no significant differences were observed amongst the other treatments. At the end of the measurement, the longest vine length was observed in T3, which reached 268.17 cm, 28.24 cm longer than the shortest treatment, T7.

As shown in Fig 5b, with the progression of the growth period, the vine thickness of netted melon continuously increased. T2, T5 and T8, which used the three-vine three-fruit pruning method, showed a smaller increase in vine thickness throughout the growth period. CK treatment had thicker vines than the three-vine three-fruit pruning treatments after May 8, but it was still thinner than in the other treatments. CK treatment showed a rapid increase in vine thickness before May 15, during the female flower blooming period, after which the growth rate slowed and became more uniform. The double-vine two-fruit treatments showed a pattern similar to that of CK, with a decrease in the growth rate after May 15. When vine thickness was measured, the order of vine thickness from thinnest to thickest was as follows: T2 < T5 < T8 < CK < T3 < T6 < T9 < T1 < T4 < T7. Under the same pruning method, vine thickness increased with plant spacing. When plant spacing was kept constant, the treatments followed the order double-vine two-fruit > three-vine two-fruit > three-vine three-fruit. This finding indicated that as the number of vines and fruits increased, the vine thickness was suppressed.

#### 3.2.2. *Growth changes in leaf number and leaf area.*

As shown in Fig 5c, the leaf number in CK treatment exhibited a steady, nearly linear increase throughout the growth period. By contrast, the leaf number in the other treatments followed a similar growth trend as the vine length, with a notable increase between May 22 and June 5. Throughout the entire growth period, CK treatment consistently had fewer leaves than the other treatments. The leaf number in the double-vine two-fruit treatments remained lower than in the other treatments (excluding CK) after June 5. At the end of the measurement, the order of leaf number from least to most was as follows: CK < T1 < T4 < T7 < T2 < T5 < T8 < T3 < T6 < T9. When the pruning method and fruit retention were the same, the leaf number increased with plant spacing. When plant spacing was consistent, an increase in the number of vines led to an increase in leaf number.

As shown in Fig 5d, the expansion of leaf area in each treatment increased rapidly before May 22, after which the growth rate slowed and became more uniform. Throughout the entire growth period, CK treatment consistently had a larger leaf area than the other treatments, whereas the treatment with a plant spacing of 55 cm generally had a smaller leaf area. At the end of the measurement, the leaf area in CK treatment was the largest at 235.13 cm², which was 1.68 times greater than the smallest leaf area in T3.

### 3.3. *Effect of different plant spacing and pruning methods on the yield of netted melon*

**Fig 6:**
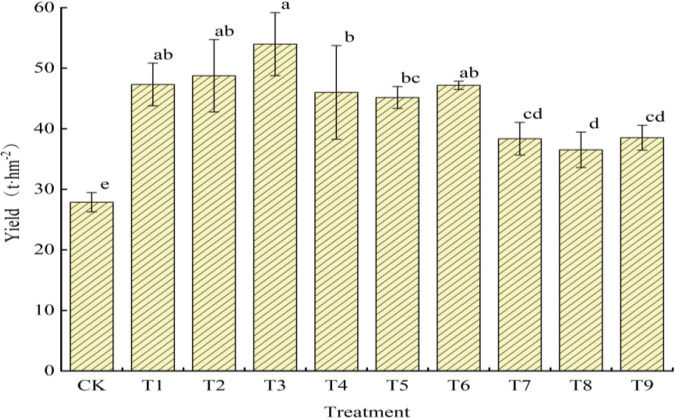
The effect of different plant spacing and pruning methods on the yield of netted melon. Note: Different letters indicate significant differences at the level of *p* < 0.05.

As shown in Fig 6, significant differences in yield can be observed amongst the treatments. The yield rankings of the treatments were as follows: T3 > T2 > T1 > T6 > T4 > T5 > T9 > T7 > T8 > CK. The highest yield was achieved by T3, with 53.97 t·hm ⁻ ², and the lowest yield was recorded in CK treatment at 27.86 t·hm ⁻ ², which was significantly lower than those in the other treatments, showing a difference of 26.12 t·hm, nearly two times that of T3. Under the same pruning method, the treatments followed the yield pattern of 55 cm > 65 cm > 75 cm in terms of plant spacing, indicating that yield decreased with increasing plant spacing. When the plant spacing was 55 cm, the yield increased with the number of retained vines. No significant difference was found between T1 and T2, which retained two fruits. However, the treatment that retained three vines, T3, with fewer retained fruits, had a higher yield than T2. At the plant spacings of 65 and 75 cm, no significant differences can be observed in terms of yield between the treatments, but all treatments followed the pattern of three-vine two-fruit > double-vine two-fruit > three-vine three-fruit. Therefore, regardless of the plant spacing, the three-vine two-fruit method consistently resulted in a higher yield than the other pruning methods.

### 3.4. Effect of different plant spacing and pruning methods on the quality of netted melon

As shown in Table 4, different plant spacing and pruning methods significantly impacted the internal and external quality of netted melons. The single fruit weight of T3 was significantly higher than that of other treatments, reaching 2.79 kg, and the single fruit weight of T2 was significantly lower than that of the other treatments, decreasing by 0.80 kg compared with T3. CK and T4 had single fruit weights above 2.5 kg, with other treatments showing no significant differences (fruit weights ranging from 2.13 kg to 2.39 kg). The highest fruit shape index was observed in CK and T3 at 1.45. As the plant spacing increased, the fruit shape index decreased regardless of the pruning method used. Under the same plant spacing, no significant differences were noted amongst the treatments. The highest flesh moisture content was found in T3 at 89.39%, which was 4.89% higher than the lowest moisture content in T5. Plant spacing did not have a significant effect on flesh moisture content. Under the same plant spacing, the pruning method did not significantly affect the flesh moisture content, but the treatments consistently followed the order three-vine two-fruit > double-vine two-fruit > three-vine three-fruit. This finding suggests that using appropriate plant spacing in the three-vine two-fruit pruning method can improve the flesh moisture content. The highest fruit firmness was observed in T8, followed by T4, with the lowest firmness found in T1, which had a firmness of 2.17. T6 had the thickest flesh at 41.46 mm, significantly higher than the other treatments. T1 had the thinnest flesh, with a thickness of 34.39 mm, and except for T1 and T6, no significant differences were found in flesh thickness amongst the other treatments.

**Table 4:** The effect of different plant spacing and pruning methods on the quality of netted melon. Note: Different letters indicate significant differences at the level of p < 0.05.

Treatments	Single fruit weight (kg)	Fruit shape index	Moisture content of fruit pulp (%)	Hardness (N)	Flesh thickness (mm)	Soluble solids content (%)	Soluble sugar content (mg/g)	Soluble sugar (%)	Vitamin C content (mg·kg^-1^)
CK	2.51 ± 0.08b	1.45 ± 0.03a	89.14 ± 0.40a	2.63 ± 0.32abc	37.49 ± 0.59abc	14.13 ± 0.64ab	1.02 ± 0.03 cd	13.43 ± 0.14c	3.15 ± 0.86bc
T1	2.37 ± 0.12bc	1.41 ± 0.12ab	88.78 ± 0.25a	2.17 ± 0.12d	34.39 ± 2.21c	13.50 ± 1.57ab	0.97 ± 0.05d	12.58 ± 0.92d	2.54 ± 0.12 cd
T2	1.99 ± 0.17d	1.39 ± 0.02abc	87.65 ± 1.25ab	2.43 ± 0.12bcd	39.25 ± 1.86abc	13.10 ± 0.66ab	0.95 ± 0.06d	12.48 ± 0.33d	2.41 ± 0.07d
T3	2.79 ± 0.13a	1.45 ± 0.05a	89.39 ± 1.74a	2.50 ± 0.10abcd	37.35 ± 3.94abc	12.73 ± 0.06b	0.98 ± 0.04d	12.97 ± 0.22 cd	2.71 ± 0.20 cd
T4	2.51 ± 0.13b	1.34 ± 0.11abc	87.87 ± 2.77ab	2.80 ± 0.26ab	40.28 ± 1.04ab	14.03 ± 1.30ab	1.04 ± 0.05 cd	13.52 ± 0.66c	3.65 ± 0.33b
T5	2.13 ± 0.23 cd	1.35 ± 0.03abc	84.50 ± 5.19b	2.37 ± 0.32 cd	34.67 ± 2.27bc	12.87 ± 0.85b	1.03 ± 0.11 cd	13.47 ± 0.16c	3.54 ± 0.14b
T6	2.39 ± 0.22bc	1.34 ± 0.05abc	87.98 ± 1.06ab	2.50 ± 0.17abcd	41.46 ± 1.59a	13.27 ± 0.81ab	1.14 ± 0.12bc	13.65 ± 0.44bc	3.69 ± 0.31b
T7	2.32 ± 0.16bc	1.27 ± 0.06c	88.53 ± 0.82ab	2.73 ± 0.31abc	37.26 ± 3.55abc	14.73 ± 1.10a	1.25 ± 0.03ab	14.39 ± 0.36ab	4.45 ± 0.35a
T8	2.21 ± 0.09 cd	1.30 ± 0.09bc	88.37 ± 1.33ab	2.87 ± 0.15a	40.17 ± 5.66ab	14.30 ± 0.62ab	1.21 ± 0.04ab	14.36 ± 0.27ab	4.43 ± 0.24a
T9	2.29 ± 0.11bc	1.28 ± 0.05bc	89.11 ± 1.86a	2.60 ± 0.10abc	35.16 ± 3.23bc	14.83 ± 0.15a	1.32 ± 0.09a	14.61 ± 0.09a	4.49 ± 0.34a

T9 had the highest levels of soluble solids, soluble protein, soluble sugars and vitamin C content. Under the same pruning method, no significant differences were noted between the treatments, but the soluble solid content was higher in the treatments with plant spacing of 75 cm than in other treatments. The treatment with the lowest soluble protein content was T3, at 0.95 mg/L. Under the same pruning method, the soluble protein content increased with plant spacing. Whilst no significant differences were found between the treatments at the same plant spacing, the order was as follows: three-vine two-fruit > double-vine two-fruit > three-vine three-fruit. The response patterns for soluble sugars and vitamin C content in netted melon under different plant spacing and pruning methods were consistent with those for soluble protein content, with higher levels observed as the plant spacing increased. When the plant spacing was consistent, the order was three-vine two-fruit > double-vine two-fruit > three-vine three-fruit. However, no significant effects of the pruning method can be observed on the soluble sugars and vitamin C content. Therefore, when two fruits are retained, increasing the number of vines and plant spacing results in the best internal quality.

### 3.5. Entropy weight TOPSIS evaluation of netted melon fruit quality indicators

After the various quality indicators of netted melon were normalized using the sum of squares method, the entropy-weight TOPSIS model was applied to obtain the comprehensive quality closeness degree (Ci) for each treatment. The results are shown in Table 5. A significant difference was found in the Ci values amongst the treatments. The comprehensive quality of the fruits, ranked from highest to lowest, was as follows: T9 > T7 > T8 > T6 > T4 > T5 > CK > T3 > T1 > T2. T9 had the highest comprehensive quality, with a Ci value of 0.935, followed by T7 and T8, with Ci values of 0.933 and 0.916, respectively. By contrast, the single-vine single-fruit treatment (CK) showed a comprehensive quality better than that of three other treatments at the 55 cm plant spacing only. This finding indicated that increasing plant density improves the comprehensive quality of netted melon. When the plant spacing was consistent, the treatments followed the order three-vine two-fruit > double-vine two-fruit > three-vine three-fruit, suggesting that the three-vine two-fruit pruning method resulted in the best comprehensive quality under the experimental conditions.

**Table 5:** Comprehensive evaluation and ranking of fruit quality of each treatment based on entropy-weighted TOPSIS method. Note: D_i_^+^ represents the distance from the optimal solution, D_i_^-^ represents the distance from the worst solution, and C_i_ represents the fit with the optimal solution.

Treatments	D_i_^+^	D_i_^-^	C_i_	Sorting Results
CK	0.214	0.119	0.357	7
T1	0.310	0.026	0.077	9
T2	0.331	0.010	0.029	10
T3	0.283	0.058	0.170	8
T4	0.137	0.197	0.589	5
T5	0.157	0.177	0.530	6
T6	0.129	0.203	0.611	4
T7	0.023	0.322	0.933	2
T8	0.029	0.319	0.916	3
T9	0.023	0.330	0.935	1

### 3.6. Optimization of planting pattern based on BP-NSGA-II model

**Fig 7:**
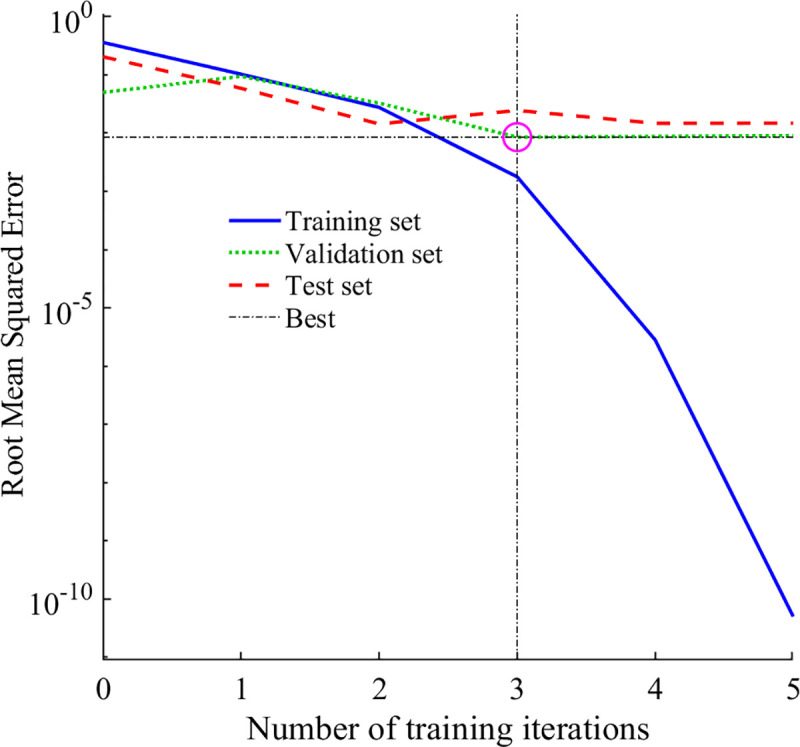
Root mean square error change plot.

**Fig 8:**
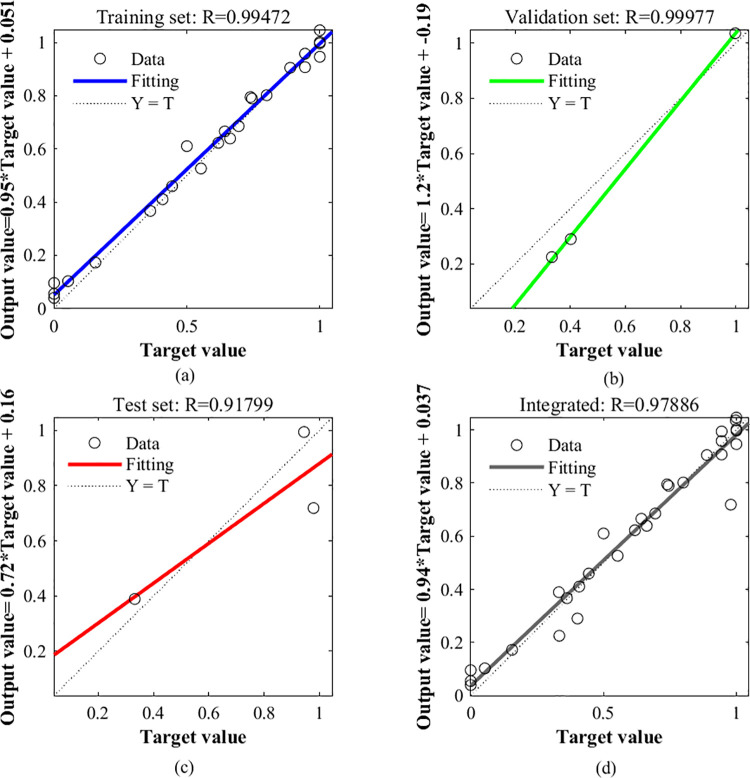
Model regression capability diagram. Note: R is the goodness of fit, and the closer it is to 1 indicates the better the fit; Y = T: Output value **(Y)** = target value **(T)** ×1; a-training set, b-validation set, c-test set, d-synthesis.

By inputting the plant spacing and pruning method of netted melon into the BP neural network and using the total growth period yield and comprehensive quality Ci as the network outputs, the MSE change after BP neural network training is shown in Fig 7, and the regression of each dataset is shown in Fig 8. As seen in Fig 7, after the model was fully trained for three iterations, the validation set reached the minimum MSE. In Fig 8, the R values for the training, testing and validation sets were greater than 0.9, indicating that the fitting model has a high degree of linearization and the results are good.

**Fig 9:**
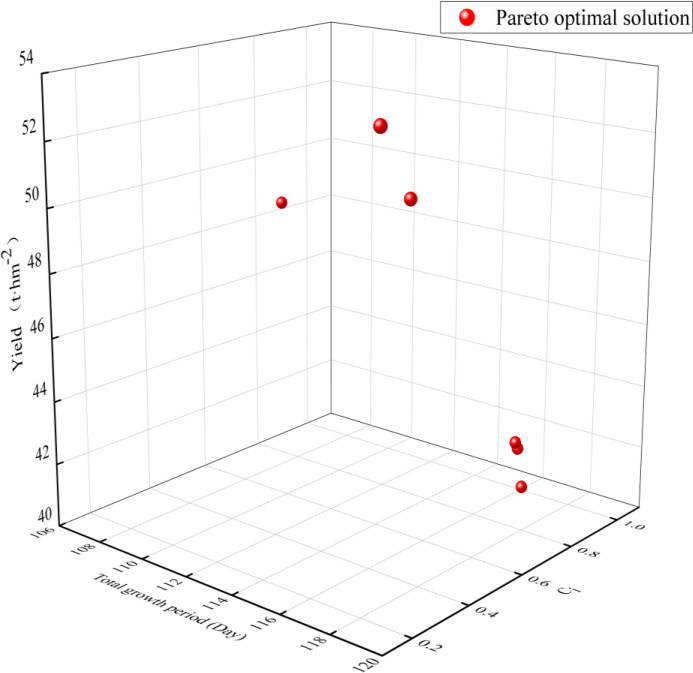
Pareto optimal solution set.

When optimizing with NSGA-II, the constraints for the plant spacing of netted melon are set on the basis of the current cultivation models [43,44]. The minimum plant spacing is 30 cm, the maximum is 80 cm and the plant spacing must be an integer divisible by 5. The constraints for pruning and fruit retention methods include four categories: single-vine single fruit, double-vine two fruits, three-vine three fruits and three-vine two fruits, which are assigned values of 1, 2, 3, and 4, respectively, in the algorithm. The multi-objective optimization targets in this study were to minimize the total growth period, maximize the yield and maximize the comprehensive quality C_i_. The Pareto optimal solution set for the total growth period, yield and C_i_ value obtained from the optimization is shown in Fig 9. Amongst the six optimal solutions, five of the pruning and fruit retention methods were three-vine two fruits, and the remaining one was double-vine two fruits. This finding suggests that in arid regions, netted melon can adopt a multi-vine multi-fruit pruning and fruit retention method.

Due to the presence of six solutions in the Pareto optimal solution set, a single optimal cultivation model for netted melon was selected to optimize the three objectives. A weighted sum method [45] was used, with equal weights (1) assigned to each of the three objectives. The optimal combination of plant spacing and pruning method was identified as a plant spacing of 70 cm and a double-vine two-fruit pruning method. Under this cultivation model, the total growth period was 107 days, the yield was 48.68 t·hm^−^² and the comprehensive quality C_i_ was 0.80.

## 4. Discussion

Reasonably adjusting planting density in combination with optimizing pruning and fruit retention methods can effectively promote crop growth [8,46]. Under the conditions of this experiment, the single-vine single-fruit CK treatment entered the fruit expansion stage the earliest, had the shortest fruit maturity period, matured the earliest and had the shortest total growth period. By contrast, the multi-vine multi-fruit pruning and fruit retention methods, which serve as direct measures to increase sources and sinks, delayed the development of melon plants, to some extent, as the number of vines and fruits increased, thereby extending the harvest time [47]. The results of the present study indicated that different plant spacing had no significant effect on fruit maturity time and total growth period across treatments. However, Díaz et al. [48] found in their study on Honey Dew melons that planting density was significantly positively correlated with the initial harvest time, meaning higher planting density delayed the harvest. This discrepancy may be attributed to the fact that the experimental variety used in the present study was netted melon, which exhibits different growth and developmental characteristics under the same planting conditions compared with Honey Dew melons [11].

In this study, the vine length exhibited an ‘S’-shaped growth curve, which is associated with the degree of physiological senescence. The growth rate of melon vines is generally fast, with rapid increases in plant height during the early stages of growth [49]. However, as senescence intensifies, the growth of vines slows down [10,50]. The vine length in T3 was significantly higher than in other treatments starting from June 12 due to the lower plant density setting favoring vine elongation [7]. This study also found that increasing plant spacing promoted vine thickness in netted melon, which is consistent with previous reports [6,51]. In this experiment, increasing the number of retained vines and fruits inhibited the expansion of vine thickness, which aligns with the findings of Fan [47] and De et al. [52]. Netted melon leaves, as the largest source in the plant, play a crucial role in maintaining the balance between vegetative and reproductive growth [13]. Appropriate plant spacing and pruning methods can promote leaf growth [53–55]. The results of the present study suggest that increasing plant spacing and the number of retained vines leads to an increase in the number of leaves because wider plant spacing reduces competition between plants, which benefits their growth [56,57]. Moreover, increasing the number of retained vines enhances the plant’s nutrient absorption and transport capacity, resulting in more leaves [58]. However, Oga et al. [46] reported that single-vine treatments had more leaves than multiple-vine treatments, because their study used smaller plant spacings, where the single-vine treatment concentrated nutrients on the main vine, promoting vegetative growth [59]. In the present study, CK treatment had a higher leaf area throughout the growth period than the other treatments, and the treatments with plant spacing of 55 cm had a generally lower leaf area than the other treatments. This finding is consistent with the findings of previous studies [46]. However, the impact of plant spacing on leaf area remains debatable. Devi et al. [25] pointed out that different planting densities do not significantly affect leaf area, and Lim et al. [6] found that lower planting densities resulted in smaller leaf areas. This finding could be due to the significant interaction between environmental conditions and genotype affecting melon growth [12], with different experimental environments and plant varieties influencing these effects.

The source–sink theory suggests that the yield and quality of crops depend on the coordination and balance between the source (photosynthetic production) and sink (fruit or storage organs). Therefore, adjusting the source–sink relationship by setting appropriate plant spacing and pruning methods plays a crucial role in influencing fruit yield and nutritional quality [60]. M. Ningdalli et al. [54] pointed out that the single-vine single-fruit pruning method helps increase the single fruit weight and overall yield of melons. Similar results were obtained under the conditions of this experiment, where the single fruit weight of CK treatment (single-vine single fruit) was 2.51 kg, lower than that of T3. However, CK treatment had the lowest yield of 27.86 t·hm ⁻ ², only because all other treatments retained ≥ two fruits. Although the single-vine single-fruit method reduced the fruit load on the plant and increased individual fruit weight, it could not compensate for the lower number of melons that could be harvested per unit area. The results of this study indicated that plant spacing affects yield, with yield decreasing as plant spacing increases. This finding is consistent with those of previous reports [9,61]. In Fan [47] study on the ‘Zhong Tian Cui Yu’ melon variety, the three-vine two-fruit pruning method enabled high-yield melon cultivation. Similar results were obtained in the present study, where the three-vine two-fruit treatment yielded better than the other pruning methods. However, other studies reported that the double-vine two-fruit pruning method resulted in the highest yield [4], whereas Ningdalli et al. [54] suggested that the single-vine single-fruit method achieved the highest yield. Therefore, in melon cultivation, choosing an appropriate pruning method based on the specific variety and growing environment is important to achieve high yields [62].

Netted melon has numerous fruit quality indicators, and analyzing individual indicators alone cannot comprehensively reflect the overall quality of the fruit. Lu et al. [63] found that the fruit comprehensive nutritional quality evaluation system constructed using the entropy-weight TOPSIS model correlated well with rankings based on single quality indicators, offering an enhanced strategy to assess overall fruit nutritional quality. Therefore, in the present study, the entropy-weight TOPSIS method was used to evaluate the overall fruit quality of netted melon. The results revealed that T9 had the best overall quality, followed by T7 and T8. This study also pointed out that increasing plant spacing improves the overall quality of netted melon, which is consistent with findings in numerous other studies [6,48,57,64]. However, Campagnol et al. [4] noted in their study on mini-watermelons in greenhouses that regardless of planting density, the fruit quality met the commercial standards, possibly due to the more precise management practices in facility cultivation than in field cultivation. The results of the present study showed that the three-vine two-fruit pruning method achieved better overall quality than the other pruning methods, aligning with Fan’s conclusion [47].

Optimizing a single objective should not be the sole focus when seeking the optimal planting model. Instead, factors closely related to economic benefits, such as early harvesting, high yield and quality must be considered comprehensively. Therefore, in the production of netted melon in arid regions, an optimization model that targets total growth period, yield and comprehensive quality should be established. The BP-NSGA-II model has already been validated by previous research [65,66] for multi-objective optimization, although it has rarely been reported in agricultural applications. In the present study, the BP-NSGA-II model was innovatively used to construct and solve a multi-objective optimization problem in the optimization of planting models. The results showed that when the plant spacing is 70 cm and the pruning method is double-vine two-fruit, the optimized total growth period is 107 days, the optimized yield is 48.68 t·hm ⁻ ² and the optimized comprehensive quality Ci is 0.80. This finding achieved the agricultural production goals of early maturity, high yield and good quality. The conclusions do not align with the optimal plant spacing and pruning methods for melons found in previous research [9,64]. The differences are likely due to the location of this study in the arid regions of Northwest China, where soil is deficient in essential mineral nutrients and the climate is relatively harsh, resulting in different growth characteristics. Considering that the model results were not validated in this study, the reliability of the optimal planting model still requires further research for confirmation.

## 5. Conclusions

(1) CK treatment, with a single-vine and single-fruit pruning method, has the shortest total growth period. This planting density and fruit retention can be used when the production goal is early harvesting. However, as the number of retained vines and fruits increases, it delays the development of melon plants and extends the harvest time.
(2) Increasing the plant spacing increases the vine thickness and leaf number but suppresses the elongation of the vine and the expansion of leaf area. Increasing the number of retained vines on netted melon plants inhibits vine thickness expansion but leads to an increase in leaf number. Similarly, increasing the number of retained fruits suppresses vine thickness expansion.
(3) T3 has the highest yield at 53.97 t·hm^−^². This planting model is recommended when the production goal is high yield. The effect of plant spacing on yield shows that yield decreases as plant spacing increases. The three-vine two-fruit treatment consistently results in higher yields than the other pruning methods.
(4) Increasing plant spacing improves the overall quality of netted melon. The three-vine two-fruit pruning method provides the best overall quality. When the production goal is high-quality melons, the planting model with T9 should be used.
(5) By using the BP-NSGA-II model for multi-objective optimization, the optimal combination was found to be a plant spacing of 70 cm and a double-vine two-fruit pruning method. According to the model, the optimized total growth period is 107 days, the optimized yield is 48.68 t hm^−²^ and the optimized fruit comprehensive quality C_i_ is 0.80. This combination achieves the agricultural production goals of early maturity, high yield and good quality.

## Supporting information

S1 Table Supplementary file - S1. (XLS)

S2 Table Tablesupplementary file - S2. (XLS)

S3 Table Tablesupplementary file - S3. (XLS)

S4 Table Tablesupplementary file - S4. (XLS)
